# Influence of age on predictiveness of genetic risk score for prostate cancer in a Chinese hospital-based biopsy cohort

**DOI:** 10.18632/oncotarget.3938

**Published:** 2015-05-15

**Authors:** Yao Zhu, Cheng-Tao Han, Hai-Tao Chen, Fang Liu, Gui-Ming Zhang, Wei-Yi Yang, Jian-Feng Xu, Ding-Wei Ye

**Affiliations:** ^1^ Department of Urology, Fudan University Shanghai Cancer Center, Shanghai, China; ^2^ Department of Oncology, Shanghai Medical Colleague, Fudan University, Shanghai, China; ^3^ Fudan Institute of Urology, Huashan Hospital, Fudan University, Shanghai, China; ^4^ State Key Laboratory of Genetic Engineering, School of Life Sciences, Fudan University, Shanghai, China; ^5^ Center for Genetic Epidemiology, School of Life Sciences, Fudan University, Shanghai, China; ^6^ Center for Cancer Genomics, Wake Forest School of Medicine, Winston-Salem, NC, USA

**Keywords:** prostate cancer, age, genetic score, predictive performance, multivariate model

## Abstract

**Background:**

We investigated whether age influences the predictiveness of genetic risk score (GRS) for prostate cancer (PCa) in a Chinese hospital-based biopsy cohort.

**Methods:**

We included consecutive patients who underwent prostate biopsies in two tertiary centers between 2012 and 2014. GRS was calculated using 24 PCa-associated genetic variants and its predictiveness was assessed by area under curve (AUC).

**Results:**

Of 1120 men tested, 724 with prostate-specific antigen (PSA) < 20 ng/ml were selected for further analysis. Patients were divided into 3 groups by age cutoffs at 60 and 70 years. GRS significantly predicted PCa for all patients (AUC: 0.561; 95% CI: 0.514–0.609) and was an independent predictor in multivariate analysis for the 60–70 year-olds (AUC: 0.612, 95% CI: 0.541–0.684), but not for patients aged < 60 years or ≥70 years. For PCa with Gleason score ≥7, GRS discriminative ability was 0.582 (95% CI=0.527–0.637) for all patients, and 0.647 (95% CI: 0.541–0.684) for the 60–70 year-old group.

**Conclusion:**

GRS significantly increased clinical prediction of PCa and high-grade disease in Chinese men aged 60–70 years, which implies that men in this age group would benefit most from genetic testing.

## INTRODUCTION

Prostate cancer (PCa) in Chinese men has rapidly increased in incidence and mortality [1]. Over the last decade, the annual percentage of change was 8% in Shanghai, and the standardized incidence rate in 2009 was 12.96 per 100,000 [1]. Traditional prostate specific antigen (PSA cutoff at 4 ng/ml had been shown to yield high false positive results in Chinese men: Na et al reported a PCa diagnosis specificity of <10% using the cutoff [2]. Therefore, arccurate prediction of PCa risk is challenging, and the cost of unnecessary biopsy is a main consideration. In consequence, a combination of tumor markers should be explorered to improve decision-making for men at risk of PCa.

Genetic risk score (GRS), derived from multiple PCa risk-associated single nucleotide polymorphisms (SNPs) have been shown to improve prostate biopsy-based diagnoses over PSA alone. Aly et al. evaluated a panel of 35 PCa risk SNPs in 5241 Swedish men who underwent prostate biopsies [3]. In multivariate analyses, GRS was an independent predictor of PCa in biopsy specimens, with an odd ratio (OR) of 1.52; discriminative ability was improved from 0.642 in a nongenetic model to 0.674 in a genetic model (*P* = 0.014). In another study, Kader et al. compared the predictive performance of 33 PCa risk SNPs with existing clinical parameters for prostate biopsies in the REDUCE trial [4]. All men in the study initially had negative prostate biopsies, and underwent study-mandated biopsies at 2 and 4 years. Overall risk of PCa was estimated and ranked for each patient in the placebo arm. The authors found GRS to be a significant predictor of PCa in multivariate analysis (OR: 1.72). Furthermore, use of GRS significantly improved the discriminative ability of the clinical model (*P* < 0.001). Similar studies conducted in Chinese men had low incidence of PCa and no PSA screening policy in place [5, 6]. Although they implied that GRS generally improved biopsy-based predictions, several drawbacks hindered its clinical application. Nearly a third of enrolled patients had PSA > 20 ng/ml. Neither study performed multivariate analyses of widely-used clinical parameters. Clinical relevant statistical measures of new markers as reclassification and net benefits were not reported [7].

Increasing age is an important risk factor for PCa [8]. A generally linear association between age and PCa incidence was observed in China [1]. Case-control reports using North American databases showed that the predictiveness of genetic scores gradually decreased in older men [9]. As prostate cancer is more likely to be life-threatening and related to hereditary factors in young men [10], we evaluated the performance of GRS by age groups to try to identify the population who would most benefit from GRS.

## RESULTS

Of 1120 consecutive men who underwent prostate biopsy, 724 had PSA < 20 ng/ml and were selected for further analyses in this study. Their median age was 66 years old (IQR: 60–72 years) and their median PSA was 8.41 ng/ml (IQR= 5.87–11.63 ng/ml). Of all enrolled patients, 24.3% were diagnosed to have PCa. Table 1 shows patients' demographic data, stratified by biopsy findings. Patients with PCa were older, had higher PSA levels, smaller prostates, abnormal DREs, and higher GRS. Family history was recorded in 2.5% of cases; it was not associated with cancer in biopsy specimen.

**Table 1:** Demographic data of enrolled patients underwent prostate biopsy (PSA < 20 ng/ml)

Variables	Stratified by biopsy outcome				*P*
	Non-Cancer		Cancer		
*n*	548		176		
Age (median [IQR])	65	[59.00, 72.00]	69	[63.00, 75.00]	<0.001
BMI (median [IQR])	23.67	[21.87, 25.39]	24.22	[22.08, 25.71]	0.361
PSA (median [IQR])	7.95	[5.40, 10.85]	10.48	[7.58, 13.75]	<0.001
Prostate volume (median [IQR])	41.6	[31.00, 56.00]	35.75	[27.55, 45.00]	<0.001
Smokers (%)	190	(43.6)	71	(47.7)	0.442
Alcohol use (%)	166	(38.1)	57	(38.3)	1
Hypertension (%)	140	(39.4)	54	(44.6)	0.37
Family history (%)	12	(2.8)	6	(4)	0.615
Diabetes mellitus (%)	37	(10.4)	16	(13.2)	0.497
DRE abnormal (%)	59	(11.7)	55	(34.2)	<0.001
Genetic risk score (median [IQR])	–0.62	[−1.03, −0.14]	–0.49	[−0.84, −0.05]	0.015

BMI: body mass index; DRE: digital rectal examination; IQR: interquartile range; PSA: prostate specific antigen.

Table 2 shows clinical characteristics and biopsy outcome stratified by age category. Patients were divided into 3 age groups by cutoffs of 60 and 70 years. As expected, PSA level, prostate volume and cancer detection rate raised gradually as age increased. Only 12.6% of men younger than 60 years old had cancer in biopsy specimen, compared with 32.3% in those older than 70 years. Interestingly, men aged 60–70 years had higher probability (68.5%) of high-grade (Gleason score ≥7) cancer. We also found older patients presented with lower BMI, healthier life styles and similar comorbidities among the 3 age groups. GRS was similar among the 3 age groups.

**Table 2:** Clinical characteristics and biopsy outcome stratified by age category

Variables	Stratified by age category						*P*
	[45,60)		[60,70)		[70,91]		
*n*	167		303		254		
Age (median [IQR])	57	[53.00, 58.00]	64	[62.00, 67.00]	75	[72.00, 78.00]	<0.001
BMI (median [IQR])	24.22	[22.49, 25.84]	24.22	[22.20, 25.90]	23.11	[21.51, 24.68]	<0.001
PSA (median [IQR])	7.38	[5.26, 10.38]	8.08	[6.06, 11.14]	9.67	[6.56, 13.08]	<0.001
Prostate volume (median [IQR])	35	[27.00, 48.00]	40.3	[31.15, 54.25]	42	[32.41, 57.47]	<0.001
Smoke = yes (%)	67	(54.9)	130	(51.8)	64	(30.2)	<0.001
Alcohol = yes (%)	59	(48.4)	105	(41.8)	59	(27.8)	<0.001
Hypertension = yes (%)	32	(33.7)	88	(42.7)	74	(42.3)	0.291
Family history = yes (%)	7	(5.7)	6	(2.4)	5	(2.4)	0.16
Diabetes mellitus = yes (%)	9	(9.5)	22	(10.7)	22	(12.6)	0.714
DRE abnormal = yes (%)	17	(11.0)	44	(15.7)	53	(23.1)	0.6
Genetic score (median [IQR])	–0.6	[−1.01, −0.15]	−0.58	[−0.99, −0.08]	−0.58	[−0.99, −0.11]	0.604
Biopsy outcome = cancer (%)	21	(12.6)	73	(24.1)	82	(32.3)	<0.001
Gleason score (%)							0.088
6	7	(33.3)	23	(31.5)	17	(20.7)	
7	11	(52.4)	20	(27.4)	43	(52.4)	
8	1	(4.8)	15	(20.5)	10	(12.2)	
9	2	(9.5)	13	(17.8)	11	(13.4)	
10	0	(0.0)	2	(2.7)	1	(1.2)	

BMI: body mass index; DRE: digital rectal examination; IQR: interquartile range; PSA: prostate specific antigen.

We first examine correlations between GRS and other risk factors of PCa such as age, PSA level, prostate volume, DRE findings and family history, but found no significant associations (Supplementary Figure 1).

In univariate analyses of candidate predictors for PCa risk by age category, GRS had moderately significant predictive value in the entire cohort (Table 3). However, this effect was attenuated in patients aged 60–70 years, in whom AUC (0.612) was close to abnormal DRE (0.623). Family history also failed to predict cancer risk. In patients younger than 60 years, none of the assessed factors were significantly associated with cancer risk.

**Table 3:** Univariate model of predictors and risk of prostate cancer stratified by age category

Predictors	Performance measurements*	Entire group	Stratified by age category		
			[45, 60)	[60, 70)	[70, 91]
Genetic risk score	AUC	**0.561**	0.556	**0.612**	0.513
	95% CI	**0.514–0.609**	0.431–0.681	**0.541–0.684**	0.438–0.588
PSA (log transformed)	AUC	**0.653**	0.604	**0.667**	**0.621**
	95% CI	**0.608–0.698**	0.483–0.726	**0.596–0.738**	**0.55–0.692**
Prostate volume	AUC	**0.615**	0.582	**0.65**	**0.65**
	95% CI	**0.566–0.663**	0.436–0.727	**0.574–0.725**	**0.578–0.722**
Family history = yes	AUC	0.506	0.503	0.506	0.514
	95% CI	0.489–0.524	0.440–0.566	0.481–0.530	0.489–0.539
DRE abnormal = yes	AUC	**0.612**	0.552	**0.623**	**0.609**
	95% CI	**0.573–0.652**	0.461–0.643	**0.562–0.683**	**0.546–0.671**

AUC: area under receiver operator characteristic curve; CI: confidence interval; DRE: digital rectal examination; PSA: prostate specific antigen.

* AUC significant different than 0.5 was in bold font

**Figure 1:**
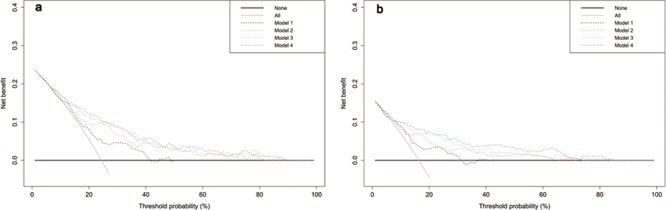
Decision curve analysis of the effect of prediction models on the detection of prostate cancer a. and high-grade disease b Net benefit is plotted against various threshold probabilities. A threshold probability indicates risk probability at which one would choose to perform a biopsy. Model 1: PSA; model 2: PSA + DRE; model 3: PSA+DRE+prostate volume; model 4: PSA+DRE+prostate volume+genetic risk score.

For patients aged 60–70 years, we constructed a multivariate model to predict cancer risk (Table 4). The multifactorial model achieved a predictive AUC of 0.786. GRS was a strong prognostic factor (OR: 0.774). Addition of GRS to a baseline model that included PSA, DRE and prostate volume significantly increased AUC and IDI. DCA showed adding GRS resulted in slightly increased net benefit (Figure 1).

**Table 4:** Multivariate analysis and increment performance of predictors and risk of prostate cancer in men aged 60–70 years

Predictors	Odds ratio	*P*	Increment in AUC	*P*	NRI	*P*	IDI	*P*
PSA (log transformed)	4.784	<0.001	0.667	-	-	-	-	-
DRE	5.548	<0.001	0.063	<0.001	0.490	<0.001	0.086	<0.001
Prostate volume	0.972	0.004	0.042	<0.001	0.532	<0.001	0.045	0.001
Genetic risk score	1.774	0.031	0.014	0.028	0.180	0.198	0.019	0.036

AUC: area under receiver operator characteristic curve; DRE: digital rectal examination; IDI: integrated discrimination improvement; NRI: net reclassification index.

Finally, we evaluated the predictive value of GRS for risk of high-grade PCa and found predictive value of GRS to be remarkably improved—particularly for the 60–70-year-old group (Supplementary Table 2), for whom GRS was an independent predictor of high-grade PCa, and significantly improved NRI and IDI (Table 5). DCA showed adding GRS to the clinical model resulted in a greater net benefit in high-grade disease (Figure 1), which was larger than the results for overall cancer risk.

**Table 5:** Multivariate analysis and increment performance of predictors and risk of high grade prostate cancer in men aged 60–70 years

Predictors	Odds ratio	*P*	Increment in AUC	*P*	NRI	*P*	IDI	*P*
PSA (log transformed)	5.549	<0.001	0.701	-	-	-	-	-
DRE	5.097	<0.001	0.039	<0.001	0.527	<0.001	0.075	0.002
Prostate volume	0.970	0.014	0.037	<0.001	0.564	<0.001	0.054	<0.001
Genetic risk score	2.752	0.002	0.028	0.001	0.365	0.022	0.050	0.006

AUC: area under receiver operator characteristic curve; DRE: digital rectal examination; IDI: integrated discrimination improvement; NRI: net reclassification index.

## DISCUSSION

This hospital-based prostate biopsy cohort confirmed the independent prognostic value of GRS for elevated risk of PCa in Chinese men. These inherited risk factors performed better in patients aged 60–70 years, and lent significant incremental improvement to the traditional multivariate model. Furthermore, GRS improved high-grade PCa risk estimation in this age group. Measurement of reclassification and net benefit support using GRS may improve clinical decision-making.

Our results have increased overall comprehension of using GRS to improve accuracy of PCa diagnosis for Chinese men. Although previous studies had already shown strong associations between GRS and PCa risk in predominately western populations [3, 4, 12], these experiences may have limited applicability in Chinese subjects. First, there are significant differences in environmental exposure as life style and dietary between residents in China and in western countries. Second, widespread use of PSA screen has induced an important age migration effect, with more men diagnosed at younger ages [13]. Third, most Chinese patients were referred with symptoms such as prostatitis or lower urinary tract symptoms, which indicate high prevalence of confounding disease in this hospital-based series. Two reports of GRS in Chinese patients found GRS was a significant predictor of biopsy outcomes [5, 6]. Jiang et al. had found the PCa detection rate in PSA < 20 ng/ml was 16.7%, 31.2% and 40.9% for men with low, average and high GRS, respectively (*P* = 0.03) [5]. Ren et al. showed GRS AUCs were 0.57 and 0.63 for PSA <10 ng/ml and 10–20 ng/ml, respectively [6]. However, these pioneer studies did not comprehensively assess additional benefit of GRS to the current clinical model, and were less patient-oriented. Also, they enrolled few patients within the PSA gray zone, which limited their applicability to the real world. Our study was designed to overcome these drawbacks, and confirmed the independent prognostic value of GRS in the prespecified age range. Moreover, the benefit of GRS was strengthened for high-grade disease, which suggests that it could help identify men who should be actively treated.

Despite several studies that showed improved cancer risk prediction by adding germline genetic markers [3, 4, 12], some results were controversial. Klein et al. showed that GRS failed to improve risk prediction based on PSA alone, either for PCa (AUC = 0.791 vs. 0.792), aggressive cancer (AUC = 0.811 vs. 0.823) or advanced stage (AUC = 0.788 vs. 0.800) [14]. The disparity may reflect differences in patient characteristics and diagnostic procedure. In Klein's study, patients were not routinely screened for PCa; thus median PSA at diagnosis was 10.7ng/ml. In population-based cohort, relatively high PSA values may be associated with high predictive performance and therefore underweight the benefit of GRS. Furthermore, the control group diagnoses relied on clinical workup, which could have contaminated results.

Our results suggested a reverse U-shape in predictive ability of genetic variants across age groups. Variation in predictive ability of genetic variants by clinical strata has also been suggested in the literature [9, 15]. In a large case-control study conducted in North America, Lindstrom et al. observed a decreasing trend in discriminative ability with advancing age (*P* = 0.009), with highest accuracy in men younger than 60 years (AUC = 0.679) [9]. These results were validated in another study, which documented a significant difference in the number of genetic variants between men diagnosed with early-onset PCa and those at older ages (*n* = 12.4 vs. 11.9, *P* <0.001) [16]. These results are paralleled in breast cancer: Aschard et al. noted decreasing discriminative ability of GRS with age (from 0.613 to 0.579 for the youngest and oldest tertile of women, respectively, *P* = 0.04) [15]. However, in our study GRS was not a significant predictor of PCa in patients <60 years old. The answer, while not yet clear, is almost certainly multifactorial. First, GRS was calculated using genetic odds ratios derived from a large genetic association study. In the report of Na et al, patients' median age was around 70 years and few patients younger than 60 years were included [11]. Therefore, differences in AUC across age strata are likely to correspond to differences in genetic effect or even different pathways across strata. Second, age group might also affect the proportion of PCa subtypes. Young patients are less likely to have Gleason ≥8 disease, than are those aged 60–70 years (14.3% vs. 41.0%). The effect of SNPs may have differential effects across PCa subtypes. Third, the sample size of current study is not adequate to detect a moderate association between GRS and PCa in younger patients.

Our results show some other interesting points as well. The prevalence of PCa is quite low (12.6%) in men younger than 60 years old with a median PSA of 7.38 ng/ml. Furthermore, of cancers in this age group, 33.3% were graded as Gleason score ≤ 6. Therefore, the probability of overdiagnosis and overtreament should be emphasized in this age group. Unfortunately, none of the examined markers provided meaningful predictive value in this study. Furthermore, family history provided limited predictive value in Chinese patients. The risk factor was not associated with biopsy-proved PCa. We also didn't observe strong association between family history and GRS.

Several limitations should be acknowledged. First, although the study was conducted in a high volume center in mainland China, the sample size is still limited for generating narrow confidence interval, defining practical cutoffs for GRS and constructing a clinical prediction tool. Second, although we have well-trained diagnostic team for prostate biopsy and pathological examination, we can rule out the possibility of a false-negative biopsy. Nevertheless, the results of this study provided additional information toward more precise prostate cancer diagnoses.

## PATIENTS AND METHODS

### Study sample

The study participants were enrolled from prospectively maintained prostate biopsy database. Briefly, consecutive patients were recruited from two tertiary hospitals in Shanghai from April 2012 until August 2014. The biopsy criteria was PSA level >4.0 ng/ml, or presence of prostate nodules detected by digital rectal examination (DRE) or ultrasound. We excluded patients with acute bacterial prostatitis diagnosed in 3 months prior to biopsy, had undergone transurethral endoscopic surgery, or had diagnoses of malignancy other than prostate adenocarcinoma. All demographic and clinical data were recorded. We only included patients with pre-biopsy PSA <20 ng/ml as this threshold had been defined as a gray zone in Chinese hospital-based series. The cancer detection rate was around a third in contemporary reports [5, 6]. This study was approved by the Institutional Review Board of Shanghai Cancer Center and Huashan Hospital, Fudan University. Written informed consent was obtained from each patient.

### Covariate and outcome definition

Before prostate biopsy, we obtained each patient's age, body mass index (BMI), PSA and prostate volume. A questionnaire was used to retrieve other clinical information as family history, comorbidities and life style. Current smokers were defined as smoking ≥100 cigarettes during their lifetime and who now smoked every day or some days. Alcohol use was defined as having at least one drink of any alcoholic beverage in the past 30 days.

As previously reported [5], GRS was calculated for each subject by their genotype at these 24 SNPs and weighted by odds ratios (ORs; detailed information in Supplementary Table 1) [11]. Briefly, (a) allelic OR for each SNP was obtained from an external study; (b) genotypic OR of each SNP was estimated from allelic OR assuming a multiplicative model; (c) risk relative to the average population risk was calculated for each genotype based on genotypic OR and genotype frequency in the Chinese population, and (d) GRS was obtained by multiplying risks relative to the population for all SNPs.

Primary outcomes were the pathological results of biopsy specimens. Transrectal ultrasound-guided biopsy using systematic scheme was performed with at least 10 cores. Pathological slides were reviewed by well-trained genitourinary pathologists.

### Statistical methods

Continuous variables were reported as median with interquartile range. Categorical variables were reported as counts with proportions. The association between continuous variables and binary outcomes was assessed using nonparametric Kruskal tests. The association between category variables and binary outcomes was assessed using chi-square tests. The relationship between GRS and continuous variables were examined using loess plots and boxplots. Logistic regression analyses were used to generate ORs and prediction models. Area under curve (AUC) was used to measure discriminative ability. The improvement in AUC was tested using likelihood ratio test. We also calculate net reclassification index (NRI), integrated discrimination improvement (IDI) and decision curve analysis (DCA) to detect information of particular clinical relevance [7]. All analyses were performed using R 3.0.1. Significance was two-sided and set at *P* < 0.05.

## CONCLUSION

In this hospital-based prostate biopsy cohort, we showed GRS significantly improved prediction of PCa and high grade disease in Chinese men aged 60–70 years. These results imply that men of this age group would benefit the most from genetic testing.

## SUPPLEMENTARY FIGURES AND TABLES


