# Supplementary material for: Influence of age on predictiveness of genetic risk score for prostate cancer in a Chinese hospital-based biopsy cohort
Source: Oncotarget. 2015 May 15;6(26):22978–84. doi: 10.18632/oncotarget.3938 (PMC4673214; doi:10.18632/oncotarget.3938)
Supplement: Supplementary file 1 [file oncotarget-06-22978-s001.pdf]

SUPPLEMENTARY FIGURE AND TABLES

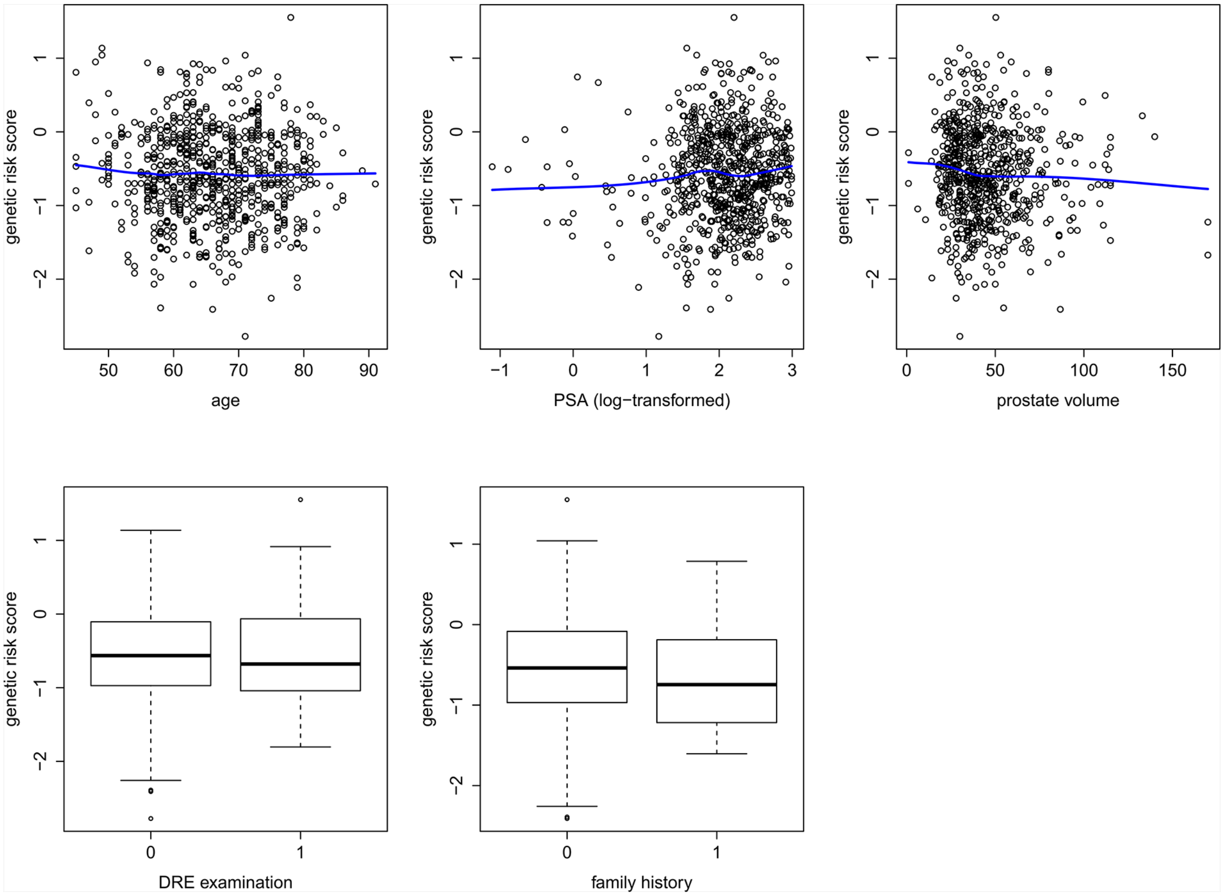

Supplementary Figure 1: Scatterplot of genetic risk score and age, PSA level and prostate volume. Blue: loess curve. Boxplot of genetic risk score and DRE findings and family history.

Supplementary Table 1: PCa risk-associated SNPs in Chinese men [9]

SNPs	Alleles	Risk alleles	Odds ratio	*P*
rs16901979	C/A	A	1.48	2.33E–14
rs1447295	C/A	A	1.48	1.54E–10
rs6983267	T/G	G	1.34	4.55E–10
rs1512268	G/A	T	1.34	8.26E–09
rs103294	T/C	C	1.34	3.15E–08
rs817826	T/C	C	1.49	8.26E–07
rs12653946	C/T	T	1.26	1.54E–06
rs9600079	G/T	T	1.24	3.50E–06
rs339331	T/C	T	1.23	2.91E–05
rs4430796	T/C	A	1.2	5.15E–04
rs620861	G/A	G	1.28	1.63E–03
rs1465618	A/G	T	1.17	3.53E–03
rs6763931	C/T	A	1.15	4.38E–03
rs2252004	G/T	C	1.17	4.42E–03
rs721048	G/A	A	1.39	1.14E–02
rs12621278	A/G	A	1.14	1.47E–02
rs11649743	C/T	G	1.11	3.15E–02
rs5759167	G/T	G	1.12	3.29E–02
rs10875943	T/C	C	1.15	3.56E–02
rs887391	T/C	T	1.1	3.66E–02
rs13385191	G/A	G	1.1	4.17E–02
rs10486567	T/C	G	1.15	4.29E–02
rs6465657	C/T	C	1.14	4.77E–02
rs9364554	C/T	C	1.1	4.83E–02

Supplementary Table 2: Univariate model of predictors and risk of high–grade prostate cancer stratified by age category

Predictors	Performance measurements*	Entire group	Stratified by age category		
			[45, 60)	[60, 70)	[70, 91]
Genetic risk score	AUC	**0.582**	0.545	**0.647**	0.539
	95% CI	**0.527–0.637**	0.390–0.699	**0.560–0.733**	0.458–0.620
PSA (log transformed)	AUC	**0.661**	0.609	**0.701**	**0.608**
	95% CI	**0.612–0.711**	0.460–0.758	**0.624–0.777**	**0.532–0.684**
Prostate volume	AUC	**0.638**	0.618	**0.666**	**0.679**
	95% CI	**0.587–0.689**	0.458–0.777	**0.584–0.748**	**0.607–0.751**
Family history = yes	AUC	0.502	0.514	0.501	0.504
	95% CI	0.486–0.519	0.433–0.595	0.476–0.525	0.483–0.525
DRE abnormal = yes	AUC	**0.631**	0.608	**0.632**	**0.622**
	95% CI	**0.585–0.677**	0.480–0.736	**0.558–0.705**	**0.554–0.689**

AUC: area under curve; CI: confidence interval; DRE: digital rectal examination; PSA: prostate specific antigen.
*AUC significant different than 0.5 was in bold font
